# Fibrinogen induces inflammatory responses via the immune activating receptor LILRA2

**DOI:** 10.3389/fimmu.2024.1435236

**Published:** 2024-09-23

**Authors:** Yifan Li, Kouyuki Hirayasu, Gen Hasegawa, Yosei Tomita, Yuko Hashikawa, Ryosuke Hiwa, Hisashi Arase, Rikinari Hanayama

**Affiliations:** ^1^ Department of Evolutionary Immunology, Advanced Preventive Medical Sciences Research Center, Kanazawa University, Kanazawa, Ishikawa, Japan; ^2^ Department of Immunology, Graduate School of Medical Sciences, Kanazawa University, Kanazawa, Ishikawa, Japan; ^3^ WPI Nano Life Science Institute (NanoLSI), Kanazawa University, Kanazawa, Ishikawa, Japan; ^4^ Department of Rheumatology and Clinical Immunology, Graduate School of Medicine, Kyoto University, Kyoto, Japan; ^5^ Department of Immunochemistry, Research Institute for Microbial Diseases, Osaka University, Suita, Osaka, Japan; ^6^ Laboratory of Immunochemistry, WPI Immunology Frontier Research Center, Osaka University, Suita, Osaka, Japan; ^7^ Center for Advanced Modalities and DDS, Osaka University, Osaka, Japan; ^8^ Center for Infectious Disease Education and Research, Osaka University, Osaka, Japan

**Keywords:** LILR, LILRA2, LILRB2, LILRA3, fibrinogen, inflammation, activating receptor

## Abstract

The leukocyte immunoglobulin-like receptor (LILR) family, a group of primate-specific immunoreceptors, is widely expressed on most immune cells and regulates immune responses through interactions with various ligands. The inhibitory type, LILRB, has been extensively studied, and many ligands, such as HLA class I, have been identified. However, the activating type, LILRA, is less understood. We have previously identified microbially cleaved immunoglobulin as a non-self-ligand for LILRA2. In this study, we identified fibrinogen as an endogenous ligand for LILRA2 using mass spectrometry. Although human plasma contains fibrinogen in abundance in its soluble form, LILRA2 only recognizes solid-phase fibrinogen. In addition to the activating LILRA2, fibrinogen was also recognized by the inhibitory LILRB2 and by soluble LILRA3. In contrast, fibrin was recognized by LILRB2 and LILRA3, but not by LILRA2. Moreover, LILRA3 bound more strongly to fibrin than to fibrinogen and blocked the LILRB2-fibrinogen/fibrin interaction. These results suggest that morphological changes in fibrinogen determine whether activating or inhibitory immune responses are induced. Upon recognizing solid-phase fibrinogen, LILRA2 activated human primary monocytes and promoted the expression of various inflammation-related genes, such as chemokines, as revealed by RNA-seq analysis. A blocking antibody against LILRA2 inhibited the fibrinogen-induced inflammatory responses, indicating that LILRA2 is the primary receptor of fibrinogen. Taken together, our findings suggest that solid-phase fibrinogen is an inflammation-inducing endogenous ligand for LILRA2, and this interaction may represent a novel therapeutic target for inflammatory diseases.

## 1 Introduction

The human leukocyte immunoglobulin (Ig)-like receptor (LILR) family consists of 13 genes, which include two pseudogenes (LILRP1 and LILRP2), five activating LILRAs (LILRA1, LILRA2, LILRA4, LILRA5, and LILRA6), five inhibitory LILRBs (LILRB1, LILRB2, LILRB3, LILRB4, and LILRB5), and one soluble LILRA3. The inhibitory LILRBs possess long intracellular regions that transduce inhibitory signals via the immunoreceptor tyrosine-based inhibitory motif (ITIM) (1, 2). The activating LILRAs, on the other hand, have a short intracellular region and bind to the FcRγ chain via a positively charged residue (arginine) in the transmembrane domain, transducing an activation signal via the immunoreceptor tyrosine-based activation motif (ITAM) (3–5). The LILR family exhibits unique expression patterns across different tissues, ranging from the immune system to the nervous system, and each LILR recognizes different ligands, potentially affecting various biological functions (6). The activation of LILRB negatively regulates acquired immunity, and the discovery of numerous ligands has revealed the significant role of LILRB in immunomodulation. HLA class I is a crucial ligand for LILRB1, and when activated, it can impede various functions, such as differentiation and antibody production in B cells (2, 7). Notably, pathogens and cancer cells evade immunity by expressing LILRB1 ligands. For instance, *Plasmodium falciparum*-infected erythrocytes express RIFIN, which binds to LILRB1 and inhibits IgM production (8). Similarly, human cytomegalovirus (HCMV)-infected cells suppress immune functions by expressing the high-affinity LILRB1 ligand UL18 (9). LILRB2 also recognizes HLA class I and inhibits myeloid cell function (10–13); it inhibits synaptogenesis by binding to Nogo66, MAG, and OMgp (14); and induces Alzheimer’s disease by binding to an oligomeric form of amyloid β (15–17). In contrast to the extensive research on LILRBs, the functions of LILRAs are not well understood, possibly due to the lack of identified ligands. LILRA2 detects microbially cleaved antibodies as non-self-ligands and is involved in immune responses against pathogens (18, 19). However, the endogenous ligands of LILRA2 remain unclear. Therefore, in this study, we aimed to identify endogenous ligands for LILRAs to gain insights into the molecular and cellular functions of the activating receptors.

## 2 Materials and methods

### 2.1 Ethics

The research ethics committee of Kanazawa University reviewed and approved this study. All the participants provided written informed consent.

### 2.2 Reporter assay

A nuclear factor of activated T cells (NFAT)-GFP reporter assay was conducted using a mouse T cell hybridoma cell line that had been genetically modified to express NFAT-GFP, FLAG-tagged DAP12, and a fusion protein comprising the extracellular domain of the target receptor and the transmembrane and intracellular domains of paired immunoglobulin-like receptor β (PILRβ) (18). The assay involved the following steps: fibrinogen or 1000-fold diluted plasma was immobilized on a 96-well plate, followed by washing with phosphate-buffered saline (PBS). The reporter cells were cultured in these wells at a density of 5 × 10^4^ cells/well at 37°C, 5% CO2 for 16 hours. In the blocking experiments, the LILRA2 reporter cells were cultured in the presence of anti-LILRA2 antibody (clone 600007, R&D Systems), anti-LILRA2 antibody (clone 135), and truncated Ig (18). In experiments with soluble fibrinogen, the binding of fibrinogen to the 96-well ELISA plate (IWAKI) was blocked by treatment with 10% BSA in advance. Then, we added the final concentration of 2 mg/mL fibrinogen and 5 × 10^4^ cells/well reporter cells and co-cultured them for 16 hours. The cells were resuspended in PBS containing 0.1% bovine serum albumin (BSA, Fujifilm) and 0.4 g/mL propidium iodide (PI, DOJINDO). The GFP expression level, which was indicative of NFAT activation, was then assessed using flow cytometry (MACSQuant Analyzer 10, Miltenyi Biotec). Data analysis was performed using FlowJo software (BD Biosciences). Fibrinogen, purified from human plasma, was obtained from Sigma–Aldrich.

### 2.3 Ligand identification

Human plasma was isolated from fresh blood by centrifugation, followed by 10-fold dilution in PBS and filtration through a 0.22μm filter to eliminate impurities. Subsequently, IgG was removed using Protein A-sepharose (GE Healthcare). IgG-free plasma was then incubated with either LILRA2 or a control (LILRB1) Fc fusion protein and immunoprecipitation was performed using Protein A-sepharose (GE Healthcare). The resulting immunoprecipitation products were eluted under acidic conditions and subjected to SDS-PAGE and Oriole staining (Bio-Rad Laboratories). The stained bands were further analyzed by in gel digestion and nano liquid chromatography (Thermo Easy-nLC1200) and mass spectrometry (Thermo Orbitrap QE plus) to identify the ligands of LILRA2.

### 2.4 Recombinant protein

Recombinant Fc fusion proteins were produced as described previously (20). Fibrinogen α-, β-, and γ-chain plasmids were constructed using the pCAGGS expression vector, and a His-tag was added to the C-terminus of the γ-chain for purifying recombinant fibrinogen. Plasmids encoding the fibrinogen α-, β- and γ-chains were transfected into 293T cells to generate the fibrinogen complex, and the resulting recombinant fibrinogen was purified using Ni Sepharose Excel (GE Healthcare). The purified recombinant fibrinogen was then confirmed in the non-reduced or dithiothreitol (DTT)-reduced form using SDS-PAGE and Coomassie Brilliant Blue (CBB) staining (Fujifilm). Fc-fusion proteins of LILRs were produced and purified as described previously (18).

### 2.5 ELISA

The binding of LILR-Fc to fibrin/fibrinogen was performed according to the standard protocol for direct enzyme-linked immunosorbent assay (ELISA). Briefly, fibrin (Sigma) and recombinant fibrinogen were immobilized in a 96-well ELISA plate (IWAKI), followed by washing with PBS-T (0.05% Tween 20 in PBS). Nonspecific binding was blocked by incubation with 1% BSA in PBS for 1 h. After washing with PBS-T, LILR-Fc was added and incubated for 2 h. After washing with PBS-T, the peroxidase AffiniPure F(ab’)_2_ fragment goat anti-human IgG Fcγ fragment-specific (Jackson) was then added, followed by TMB reagent (Nacalai) for color development. For competitive ELISA, LILRA3- or LILRB1-mouse IgG2a-Fc was added prior to adding LILRB2-Fc or LILRA2-Fc. To measure IL-8 in monocyte culture supernatants after fibrinogen stimulation, an ELISA MAX Deluxe Set for human IL-8 (BioLegend) was used according to the manufacturer’s instructions. Data were collected using a plate reader (Enspire, PerkinElmer).

### 2.6 Immune response by monocytes

Fresh blood was obtained from healthy donors. Neutrophils were isolated from fresh blood using MACSxpress Whole Blood Neutrophil Isolation Kit (Miltenyi Biotec). Peripheral blood mononuclear cells (PBMCs) were separated by centrifugation using a LeucoSep-tube (Greiner) supplemented with Ficoll (Cytiva) according to the manufacturer’s instructions. Monocytes were isolated from PBMCs using CD14 MicroBeads, human (Miltenyi Biotec), according to the manufacturer’s instructions. Monocytes were resuspended in RPMI 1640 medium supplemented with 10% fetal bovine serum (FBS) (Gibco), 1X penicillin-streptomycin solution (Wako), and 10 ng/mL human M-CSF (Peprotech). Monocytes were then added to pre-immobilized fibrinogen on 96-well plates at 3 × 10^5^ cells/well in the presence of anti-LILRA2 antibody (clone 600007, R&D Systems), or an isotype control (mouse IgG1, R&D Systems), or anti-LILRB2 antibody (clone 287219, R&D Systems), or an isotype control (mouse IgG2a, R&D Systems), and incubated for 5 h at 37°C. After centrifugation, the supernatant was collected for measuring the human IL-8 levels, and the cell pellets were subjected to RNA extraction according to the manufacturer’s protocol using RNA Plus XS (NucleoSpin) for RNA-seq analysis. To validate the findings of RNA-seq analysis, we conducted digital PCR assays targeting SERPINE1, CCL24, and TNF according to the QIAcuity digital PCR system (Qiagen). Expression levels were normalized to UBE2D2, a suitable reference gene for PBMCs (21). The primer-probe sets were obtained from Integrated DNA Technologies.

### 2.7 Analysis of fibrinogen binding to monocytes

LILRA2 on monocytes was labeled by adding anti-LILRA2 antibody (clone 600007, R&D Systems), followed by allophycocyanin (APC) AffiniPure F(ab’)_2_ fragment goat anti-mouse IgG Fcγ fragment specific (Jackson). For determining fibrinogen binding to monocytes, recombinant fibrinogen was added to the monocytes, followed by rabbit anti-human fibrinogen (Dako), and APC AffiniPure F(ab’)_2_ fragment donkey anti-rabbit IgG (H+L) (Jackson). After incubation on ice for 30 min in each staining step, the cells were washed twice and resuspended in PBS containing 0.1% BSA and 0.4 µg/mL PI (DOJINDO). The cells were analyzed using flow cytometry (MACSQuant Analyzer 10, Miltenyi Biotec) and the data were analyzed using FlowJo software (BD Biosciences).

### 2.8 RNA-seq analysis

The monocyte-derived mRNA library was constructed and sequenced by the Beijing Genomics Institute (BGI) using the DNBSEQ platform with 150 bp paired-end reads. Sequencing data were filtered using the SOAPnuke (BGI) to remove adaptors and low-quality reads. After obtaining clean reads, HISAT was used to map the clean reads to the reference genome (GRCh38.p13), and Bowtie2 was used to align the clean reads to reference genes. Gene expression from RNA-Seq data was analyzed using TCC-GUI and visualized using a volcano plot (22). Gene set enrichment analysis (GSEA) was performed using Broad Institute Software (23, 24).

## 3 Results

### 3.1 Identification of the endogenous ligand for LILRA2

**Figure 1:**
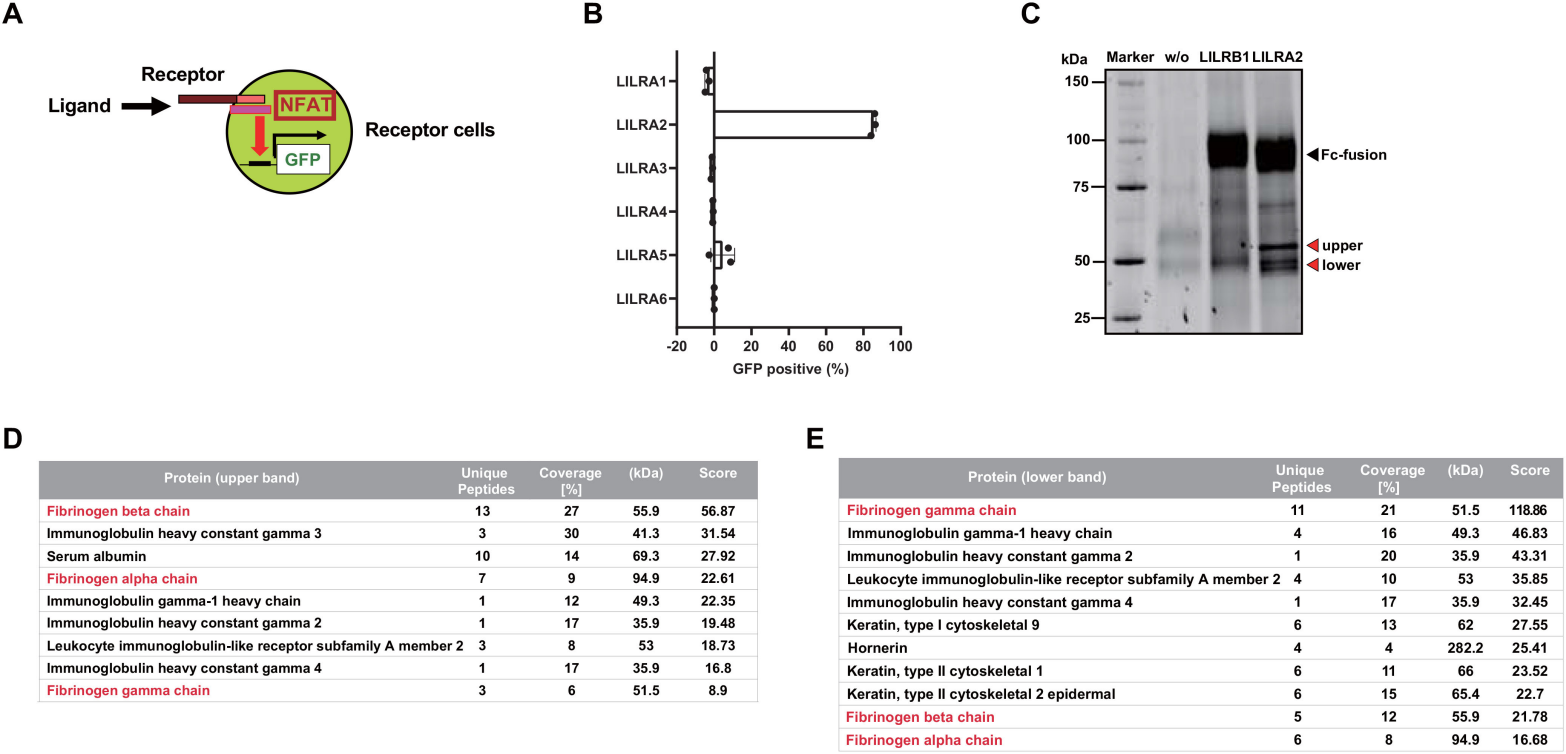
Identification of the endogenous ligand for LILRA2. **(A)** Schematic diagram of NFAT-GFP reporter cells, which express GFP upon ligand recognition. **(B)** LILRA1-6 reporter cells were co-cultured with human plasma and GFP expression was examined using flow cytometry. Percentages of GFP-positive cells were presented by subtracting those of non-treated from treated cells. **(C)** Human plasma was immunoprecipitated with LILRA2-Fc or control (LILRB1)-Fc and analyzed using SDS–PAGE and Oriole staining of the immunoprecipitated product. w/o indicates human plasma without Fc-fusion protein. Fc-fusion band (black triangle), ligand candidate band (red triangle). **(D, E)** A list of protein components obtained from the ligand candidate bands (upper band **(D)**, lower band **(E)**) using mass spectrometry analysis. The protein names are arranged in the descending order of their scores. Data are representative of at least three independent experiments, except for **(C–E)**, which were performed once.

To search for potential ligands that activate LILRAs, we generated NFAT-GFP reporter cells expressing the extracellular domains of LILRAs, which induce GFP upon ligand recognition (**Figure 1A**) (25). In the process of screening ligand-containing materials, LILRA2 reporter cells induced GFP expression when co-cultured with human plasma, whereas other LILRA reporter cells did not (**Figure 1B**). This result indicated the presence of a LILRA2-specific ligand in human plasma. To identify this LILRA2 ligand, we performed an immunoprecipitation analysis using LILRA2-Fc and human plasma. SDS-PAGE and Oriole staining revealed that some specific bands of approximately 50 kDa specifically co-immunoprecipitated with LILRA2-Fc but not LILRB1-Fc (**Figure 1C**). To identify these protein bands, one band larger than 50 kDa (upper band) and two bands smaller than 50 kDa (lower band) were excised and subjected to mass spectrometry (**Figure 1C**). The highest scores of the upper and lower bands were found for fibrinogen beta and gamma, respectively (**Figures 1D, E**), suggesting that fibrinogen is the LILRA2 ligand candidate.

### 3.2 Validation of fibrinogen as the LILRA2 ligand

**Figure 2:**
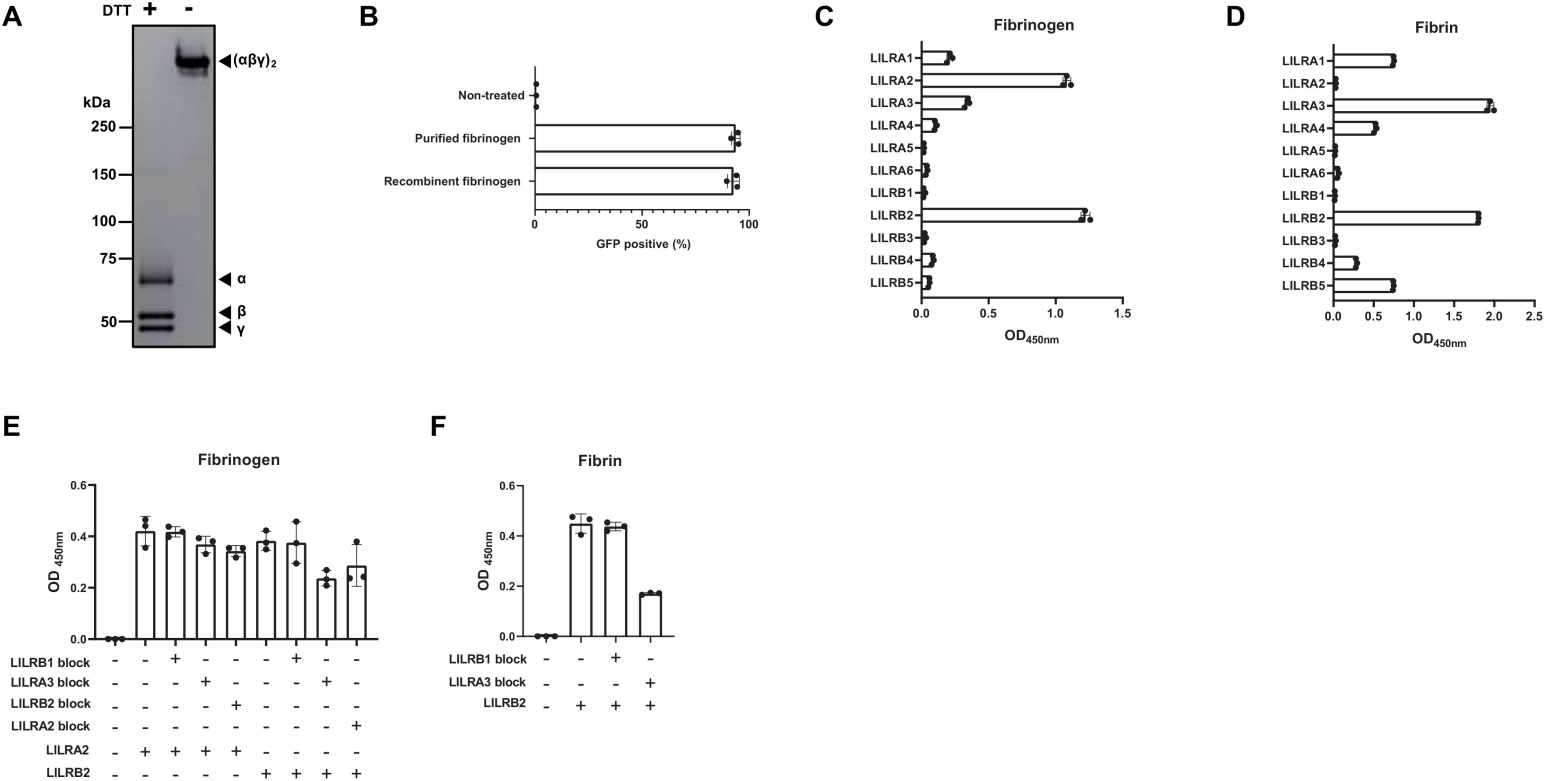
Validation of fibrinogen as the ligand for LILRA2. **(A)** Recombinant fibrinogen (non-reduced and DTT-reduced) was visualized by SDS-PAGE and CBB staining. **(B)** LILRA2 reporter cells were co-cultured with commercially available purified fibrinogen and with recombinant fibrinogen; GFP expression was then examined by flow cytometry. **(C)** Binding of LILR-Fc to fibrinogen coated on the ELISA plate. **(D)** Binding of LILR-Fc to fibrin coated on the ELISA plate. **(E, F)** Binding of LILRA2-, or LILRB2-human IgG Fc to fibrinogen **(E)**, or fibrin **(F)** in the presence of LILRA3-mouse IgG2a Fc or control-mouse IgG2a Fc (LILRB1), as measured using ELISA. Data are representative of at least three independent experiments **(A–C)**, or two independent experiments **(D–F)**.

Fibrinogen is a glycoprotein with a molecular weight of 340 kDa and a concentration of 2–4 mg/mL in human plasma. It consists of two α-chains (63.5 kDa), two β-chains (56 kDa), and two γ-chains (47 kDa) connected by disulfide bonds (26). Thrombin converts fibrinogen to a fibrin matrix and plays an important role in hemostasis (27). To determine whether fibrinogen is a ligand for LILRA2, we generated recombinant fibrinogen by co-transfecting the plasmids encoding fibrinogen α-, β-, and γ-chains into 293T cells, followed by His-tag purification. SDS-PAGE and CBB staining of recombinant fibrinogen confirmed that all the chains (α-, β-, and γ-chain) appeared at the predicted sizes under reducing conditions, and that the (αβγ)2 complex appeared at around 340 kDa under non-reducing conditions (**Figure 2A**). This recombinant fibrinogen, as well as commercially available purified fibrinogen was found to activate LILRA2 reporter cells, confirming that fibrinogen is the ligand for LILRA2 (**Figure 2B**). Next, we examined whether LILRA2 was the only fibrinogen receptor in the LILR family. To this end, we tested all members of the LILR family (A1-A6, B1-B5) using an ELISA, in which fibrinogen was coated on the assay plate. The results showed that fibrinogen bound to LILRA2 as well as to LILRB2, suggesting that LILRA2 and LILRB2 are paired activating and inhibitory receptors for fibrinogen, respectively (**Figure 2C**). Fibrinogen activated by thrombin is converted to fibrin, which may also act as a ligand for LILRA2 and LILRB2. Therefore, we examined whether fibrin could bind to LILRA2 and LILRB2 using ELISA. Notably, LILRA2 did not recognize fibrin, whereas LILRB2 as well as LILRA3 bound to fibrin (**Figure 2D**). To determine whether LILRA3, the only soluble form, hinders LILRB2 recognition by ligands, competitive ELISA was performed using LILRA3 to block the binding of LILRB2 to fibrinogen/fibrin. The results showed that LILRA3 had a blocking effect on both LILRB2-fibrinogen and LILRB2-fibrin interactions (**Figures 2E, F**). In contrast, neither soluble LILRB2 nor LILRA3 inhibited the LILRA2-fibrinogen interaction (**Figure 2E**). Taken together, these results suggest that conversion of fibrinogen to fibrin may turn off LILRA2 activation and then turn on inhibitory signals over activating signals, which are further regulated by LILRA3.

### 3.3 Solid-phase fibrinogen is recognized by LILRA2

**Figure 3:**
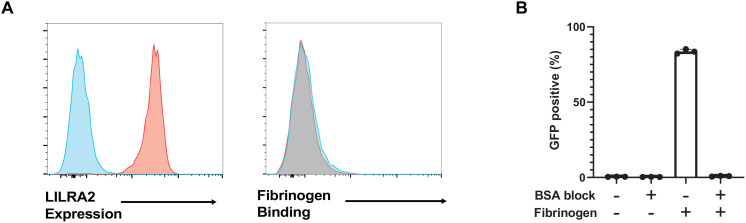
Soluble fibrinogen is not recognized by LILRA2. **(A)** The left panel shows LILRA2 expression on human monocytes. The right panel presents fibrinogen binding to human monocytes, as analyzed with soluble fibrinogen, and anti-fibrinogen antibody using flow cytometry. Blue histograms indicate the negative controls and red histograms represent LILRA2 expression as well as fibrinogen binding, respectively. **(B)** LILRA2 reporter cells were co-cultured with soluble or solid-phase fibrinogen in the presence or absence of BSA blocking. GFP expression was examined using flow cytometry. Data are representative of at least two independent experiments **(A)**, or three independent experiments **(B)**.

A large amount of soluble fibrinogen is present in plasma. However, it is unlikely that soluble fibrinogen constitutively activates LILRA2 in immune cells. Therefore, we performed binding assays using soluble fibrinogen and human primary monocytes, which show high expression of LILRA2 on their surface. Flow cytometric analysis showed that although LILRA2 was detected in human monocytes, soluble fibrinogen did not bind to monocytes (**Figure 3A**). Furthermore, we conducted the reporter assay by preventing fibrinogen binding to the plate surface by prior blocking with BSA so that the reporter cells were only exposed to soluble fibrinogen. The LILRA2 reporter assay showed that soluble fibrinogen could not activate LILRA2 (**Figure 3B**). These data suggest that fibrinogen binding to a certain target (solid-phase fibrinogen) may be necessary for fibrinogen recognition by LILRA2.

### 3.4 Immune responses against solid-phase fibrinogen by LILRA2

**Figure 4:**
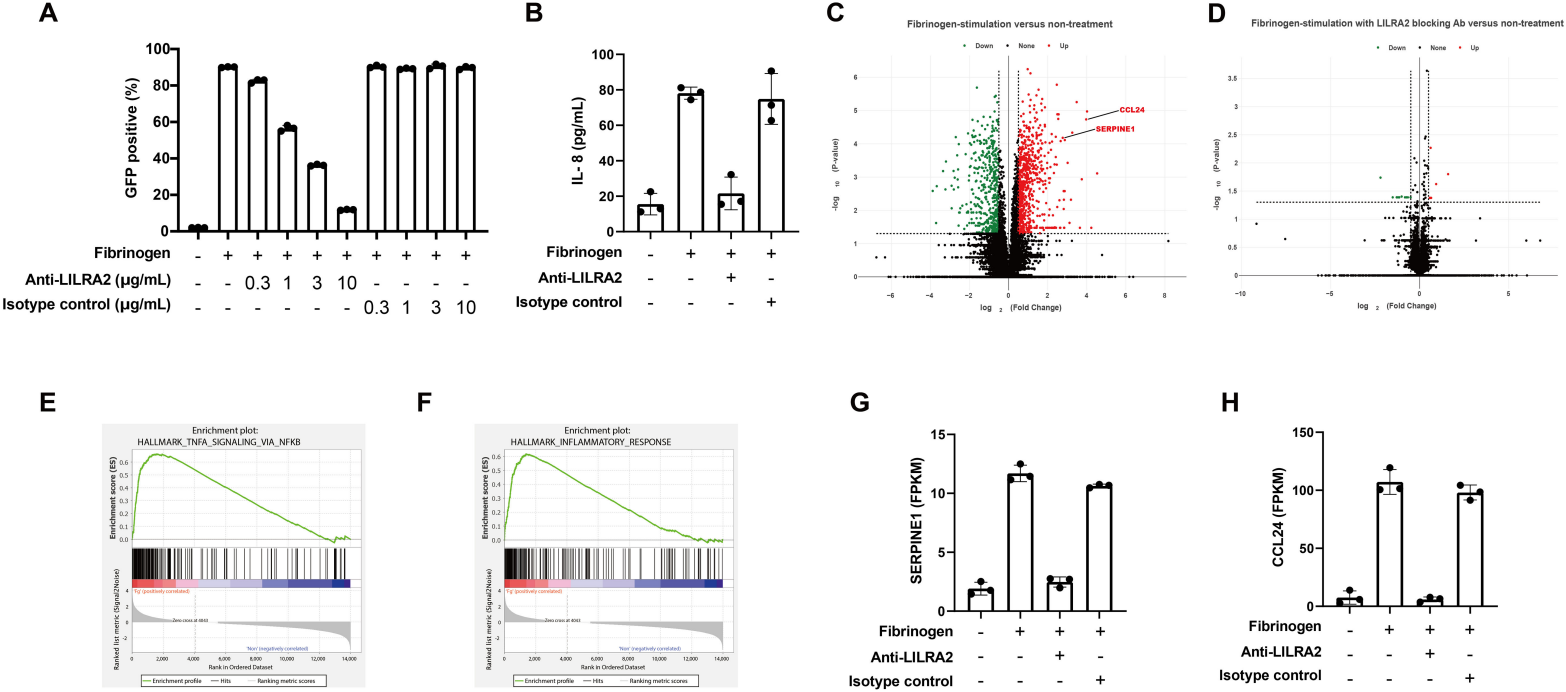
Solid-phase fibrinogen induces immune responses through LILRA2. **(A)** The blocking effect of anti-LILRA2 or the isotype control on the LILRA2-fibrinogen interaction on LILRA2 reporter cells. GFP expression was examined using flow cytometry. **(B)** Human primary monocytes were co-cultured with immobilized fibrinogen with or without LILRA2 blocking antibody. The culture supernatant for IL-8 production was examined using ELISA. **(C)** Volcano plot of differentially expressed genes (DEGs) indicating fibrinogen-stimulation versus non-treatment on human primary monocytes. Upregulated genes (Red dots) and downregulated genes (Green dots) are shown. P-value cutoff = 0.05 and fold-change cutoff = 0.5. **(D)** Volcano plot of DEGs indicating fibrinogen-stimulation with LILRA2 blocking antibody versus non-treatment. P-value cutoff = 0.05 and fold-change cutoff = 0.5. **(E, F)** Gene set enrichment analysis (GSEA) graph showing positive correlation with TNF signaling **(E)** and inflammatory response **(F)** in fibrinogen-stimulation versus non-treatment. **(G, H)** Representative changes in the gene expression of SERPINE1 **(G)**, and CCL24 **(H)** after fibrinogen-stimulation without or with LILRA2 antibody blocking. Data are representative of at least three independent experiments **(A, B)**, or one individual **(C–H)** with technical triplicates.

To investigate the effect of LILRA2 on primary monocytes recognizing solid-phase fibrinogen, we first confirmed whether a commercially available LILRA2 antibody could block the LILRA2-fibrinogen interaction. To this end, we co-cultured solid-phase fibrinogen and LILRA2 reporter cells incubated with different concentrations of the blocking antibody. The LILRA2 reporter assay showed that the anti-LILRA2 antibody (clone 600007) effectively blocked the activation of LILRA2 and that 10 µg/mL was required for sufficiently blocking the LILRA2-fibrinogen interaction (**Figure 4A**). In addition, the anti-LILRA2 antibody (clone 600007) exhibited no agonistic effects on the LILRA2 reporter cells (**Supplementary Figure 1A**). Although anti-LILRA2 antibody (clone 135) also inhibited the LILRA2-fibrinogen interaction, its blocking efficacy was slightly weaker compared to the anti-LILRA2 antibody (clone 600007) (**Supplementary Figure 1B**). In contrast, a different LILRA2 ligand, the truncated Ig, was unable to impede the LILRA2-fibrinogen interaction (**Supplementary Figure 1B**). Based on these findings, we proceeded with the anti-LILRA2 antibody (clone 600007) for subsequent analyses. To examine the effect of fibrinogen on primary cells, we co-cultured human primary monocytes with immobilized fibrinogen, with or without an anti-LILRA2 blocking antibody, and examined the culture supernatant for IL-8 production using ELISA, which was known to be induced by LILRA2 stimulation as described previously (18). As expected, immobilized fibrinogen promoted IL-8 expression in monocytes compared to that in non-treated cells (**Figure 4B**). Moreover, IL-8 expression decreased to the same level as that in untreated or isotype control groups after LILRA2 was blocked using the blocking antibody (**Figure 4B**). This result indicated that immobilized fibrinogen could activate monocytes through LILRA2, and that this activation was explained mainly by LILRA2 (**Figure 4B**). However, fibrinogen failed to induce IL-8 production in neutrophils, in contrast to its effect on monocytes (**Supplementary Figure 2A**). To address this discrepancy, we analyzed the expression of both LILRA2 and LILRB2, which constitute paired activating and inhibitory receptors for fibrinogen, respectively, in both cell types. Notably, the LILRA2/LILRB2 expression ratios were significantly lower in neutrophils than monocytes (**Supplementary Figures 2B, C**), suggesting that neutrophils exert a more potent inhibitory effect on fibrinogen-mediated responses than monocytes. To further validate our findings, we analyzed an additional cohort of three individuals in our experiments on primary monocytes. As shown in **Supplementary Figure 3**, the anti-LILRA2 antibody alone elicited moderate agonistic responses in some individual, although the degree of effectiveness exhibited variation among individuals. Nonetheless, its ability to block the interaction between LILRA2 and fibrinogen remained consistent across individuals. On the other hand, we also investigated the effect of the anti-LILRB2 antibody on primary monocytes stimulated with fibrinogen. However, the anti-LILRB2 antibody alone induced IL-8 production in primary monocytes even without fibrinogen stimulation (**Supplementary Figure 4**). This observation could be due to either the blockade of endogenous ligands for LILRB2, or the antibody’s agonistic activity. Our finding as shown in **Figure 4B** and **Supplementary Figure 3** indicated that the anti-LILRA2 antibody effectively suppressed fibrinogen-mediated immune responses, suggesting that LILRB2 is not a key player in this context. Next, to understand the gene expression in monocytes after fibrinogen stimulation, we performed RNA-seq analysis of human primary monocytes. Analysis of the RNA-seq data showed that the expression of many genes was altered by fibrinogen stimulation; however, these changes largely disappeared when LILRA2 was blocked (**Figures 4C, D**). Gene set enrichment analysis (GSEA) showed that fibrinogen stimulation was positively correlated with TNF signaling and inflammation (**Figures 4E, F**, **Supplementary Figures 5A, B**). Further, LILRA2 activation by fibrinogen induced several differentially expressed genes, such as the fibrinolytic inhibitor SERPINE1 and the CCL24 (**Figures 4G, H**, **Supplementary Figures 6A, B**). A comparison of the data with and without LILRA2 blockade showed that the pathways activated by fibrinogen stimulation were almost exclusively associated with LILRA2 activation (**Supplementary Figures 5A, B**). To validate the findings of RNA-seq analysis, we conducted digital PCR assays targeting SERPINE1, CCL24, and TNF in three individuals. We consistently confirmed the up-regulation of CCL24 and SERPINE1 following fibrinogen stimulation across individuals (**Supplementary Figure 7**). In addition, TNF expression was slightly up-regulated after fibrinogen stimulation, although there exists considerable variability in the effects of the anti-LILRA2 antibody. Taken together, our results suggest that monocytes can recognize solid-phase fibrinogen bound to certain target surfaces via LILRA2, which is involved in inflammatory responses.

## 4 Discussion

In the present study, we found that fibrinogen is a novel endogenous ligand for activating LILRA2 and inhibitory LILRB2. Moreover, fibrin, which is converted from fibrinogen, is recognized by LILRB2 and LILRA3 but not by LILRA2. These observations suggest that the ligand status determines either the activating or inhibitory effects on immune cells. Because LILRA3 is a soluble protein, LILRA3 may act as an antagonist of LILRB2, which recognizes fibrin. In fact, both LILRB2 and LILRA3 are Nogo66 receptors. LILRA3 can reverse the neuronal growth inhibition mediated by LILRB2-Nogo66 and is considered a competitive inhibitor of cell surface inhibitory receptors (28). We confirmed that LILRA3 blocked the binding of LILRB2 to fibrinogen and fibrin at the protein level. LILRA3 is characterized by a high frequency of deficiency in Northeast Asian populations, suggesting that the *LILRA3* gene has undergone positive natural selection (29, 30). Therefore, the LILRA3-fibrin axis may be involved in the selective pressure on the *LILRA3* gene. Additionally, *LILRA3* deletion with HLA-B*52 is significantly associated with Takayasu arteritis (TAK), characterized by inflammation of the aorta (31), suggesting that LILRA3-mediated blocking of the LILRB2-fibrin interaction may induce resistance to TAK. Furthermore, LILRA2 bound to solid-phase but not to soluble fibrinogen, suggesting that recognition by LILRA2 is strictly regulated by ligand structural changes. This was also observed for previously identified ligands in which microbially cleaved immunoglobulin, but not intact immunoglobulin, was recognized by LILRA2 (18). Structural analysis of the specific conformation of LILRA2-recognizing fibrinogen is thus required to elucidate the underlying recognition mechanism.

In the blood, LILRA2 is mainly expressed on neutrophils and monocytes, including classical, intermediate, and inflammatory monocyte subsets. However, fibrinogen failed to induce IL-8 production in neutrophils, unlike its effect on monocytes. This discrepancy may be partially attributed to the differential LILRA2/LILRB2 expression ratios between neutrophils and monocytes. Higher LILRA2/LILRB2 expression ratios in monocytes than neutrophils suggest that LILRA2 activation could predominate over LILRB2 inhibition in monocytes. Conversely, our understanding of LILRA2 expression in tissues remains limited. Some reports suggest that LILRA2 is also expressed in macrophages, and microglial cells (32, 33). Therefore, further investigation is warranted to understand the precise roles of LILRA2-fibrinogen interactions in distinct monocyte subsets or tissue-resident macrophages.

LILRA2 recognizes solid-phase fibrinogen, which is found under some physiological conditions. At the site of vascular injury, platelets are recruited and activated to bind fibrinogen to surface integrin receptors to generate platelet-fibrin clots. Under these conditions, fibrinogen is present on the platelet surface and may be a target of LILRA2. RNA-seq analysis showed that fibrinogen-stimulated monocytes induced high expression of the fibrinolysis inhibitor SERPINE1, suggesting that the fibrinogen status may play an important regulatory role in plasminogen activation. Therefore, LILRA2 in circulating monocytes may recognize fibrinogen-bound platelets and promote fibrin formation for hemostasis. Paradoxically, we also observed that SERPINB2 showed marked down-regulation following fibrinogen stimulation, as shown in **Supplementary Figure 6**. Both SERPINE1 and SERPINB2 belong to the serpin superfamily and function as inhibitors of plasminogen activators. However, accumulating evidence suggests that their functions extend beyond mere inhibition of plasminogen activators. Specifically, SERPINB2 may counteract the distinctive functions attributed to SERPINE1, particularly in the context of cancer prognosis (34). These observations imply that the LILRA2 signaling pathway, triggered by fibrinogen, selectively enhances SERPINE1 functions, without invoking competition from SERPINB2. Another important condition for solid-phase fibrinogen is the bacterial surface. A subset of bacteria can bind fibrinogen on the cell surface to escape from the host immune system (35–38). Thus, LILRA2 may recognize fibrinogen-bound bacteria and detect bacterial invasion. The previously identified LILRA2 ligand, a microbially cleaved immunoglobulin, is also a product of bacterial immune evasion. Therefore, LILRA2 may play an important role in opposing bacterial immune evasion.

LILRA2 SNP rs2241524 has been reported to be associated with microscopic polyangiitis (MPA) (39). MPA is a type of vasculitis characterized by the inflammation of small blood vessels (40). Therefore, the interaction between LILRA2 and fibrinogen may be involved in MPA pathogenesis. Additionally, LILRA2 expression is significantly upregulated in lesions of patients with disseminated leprosy (41). In an earlier study, LILRA2 activation by cross-linking antibodies induced a shift from IL-12 to IL-10 production in monocytes. However, LILRA2 stimulation with fibrinogen attenuated IL-10 production in monocytes in the present study. This discrepancy could be attributed to the differences between antibody and fibrinogen stimulation. Fibrinogen stimulation promoted high expression of several chemokines in monocytes, with a particularly marked increase in CCL24. CCL24 induces eosinophil chemotaxis by interacting with the chemokine receptor CCR3, and has a strong chemotactic effect on resting T lymphocytes, but only a slight chemotactic effect on neutrophils (42, 43). In addition, the increased expression of IL-8, CCL4, CCL5, and CCL22 (**Supplementary Figure 6**) suggests that multiple immune cells are involved in the inflammatory response triggered by fibrinogen and LILRA2 (44–47). It is noteworthy that CCL24 has shown potent bactericidal activity against a wide range of bacteria, including *Streptococcus pneumoniae* and *Staphylococcus aureus*, through membrane disruption (48).

Primary monocytes induced inflammatory responses to solid-phase fibrinogen. Anti-LILRA2 antibody treatment completely blocked this inflammatory pathway, indicating that LILRA2 is the principal inflammatory signal against fibrinogen. Fibrinogen is known to be involved in various diseases, including inflammatory diseases, infections, cancer, thrombotic diseases, and vascular wall diseases (49). Therefore, blocking LILRA2-fibrinogen interactions may serve as a promising therapeutic strategy for these diseases. In this regard, however, we observed the agonistic activity associated with the anti-LILRA2 antibody in some individual, which poses a challenge for the development of antibody-based therapies targeting LILRA2. Structural analysis of the LILRA2-fibrinogen complex is required to fully understand the blocking and agonistic effects of the anti-LILRA2 antibody on monocytes. Further studies are needed to elucidate the physiological role of LILRA2-fibrinogen interactions.

## Data Availability

The datasets presented in this study can be found in online repositories. The names of the repository/repositories and accession number(s) can be found below: https://www.ddbj.nig.ac.jp/, DRR550124-DRR550135.
