# Supplementary figures and images for: Fibrinogen induces inflammatory responses via the immune activating receptor LILRA2
Source: Front Immunol. 2024 Sep 23;15:1435236. doi: 10.3389/fimmu.2024.1435236 (PMC11456740; doi:10.3389/fimmu.2024.1435236)

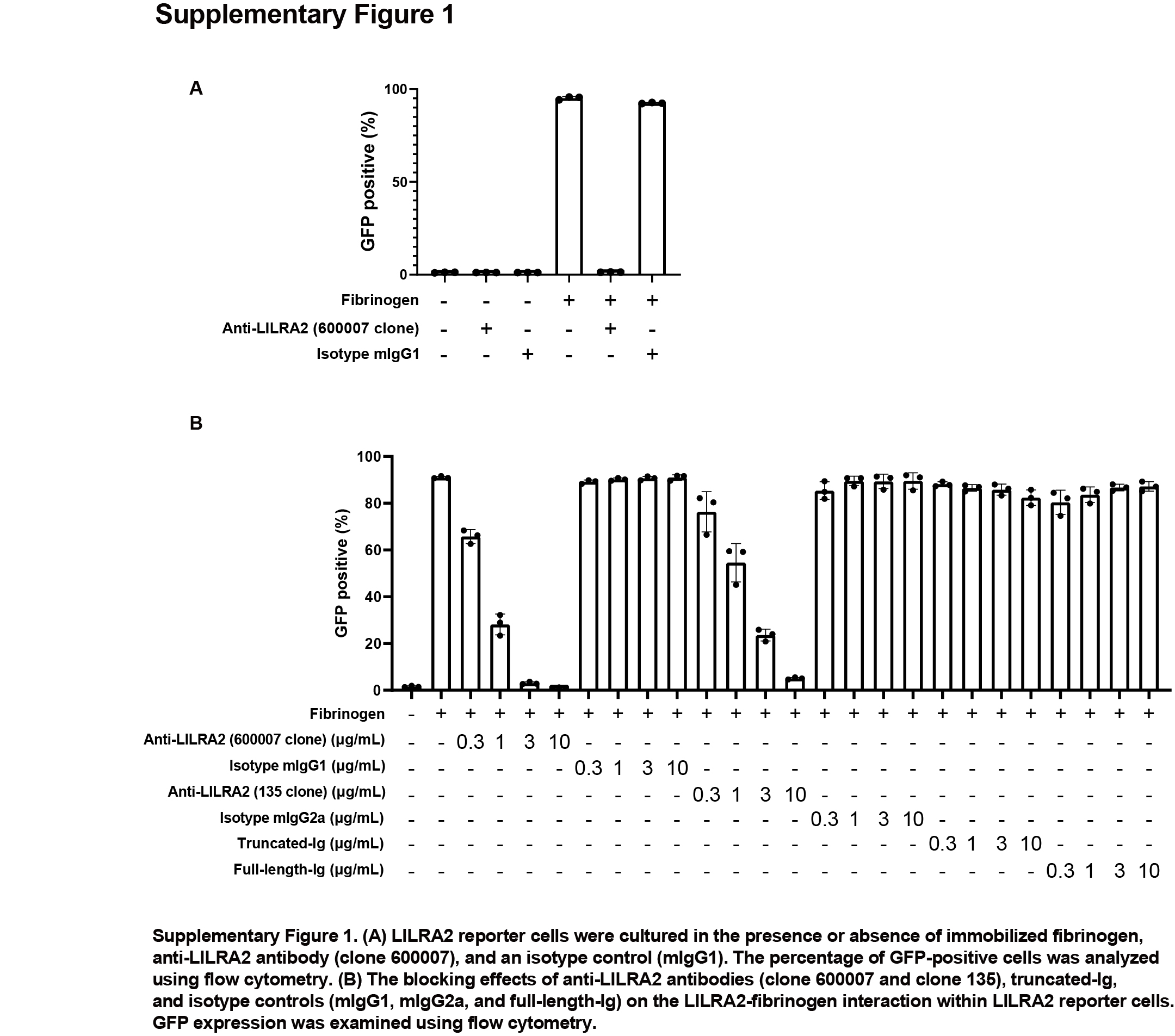

Supplement: Supplementary file 1 [file Image1.jpeg]

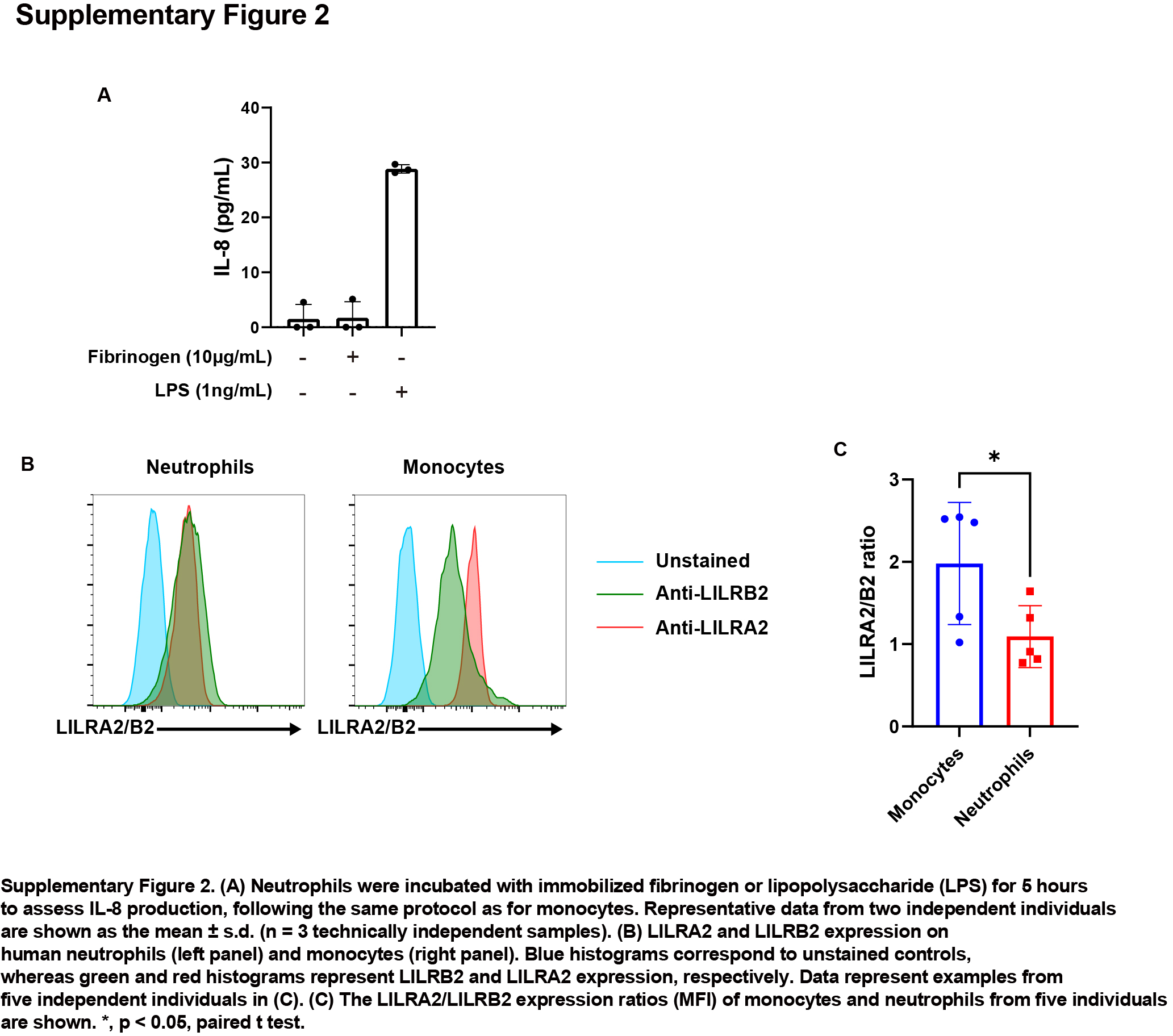

Supplement: Supplementary file 2 [file Image2.jpeg]

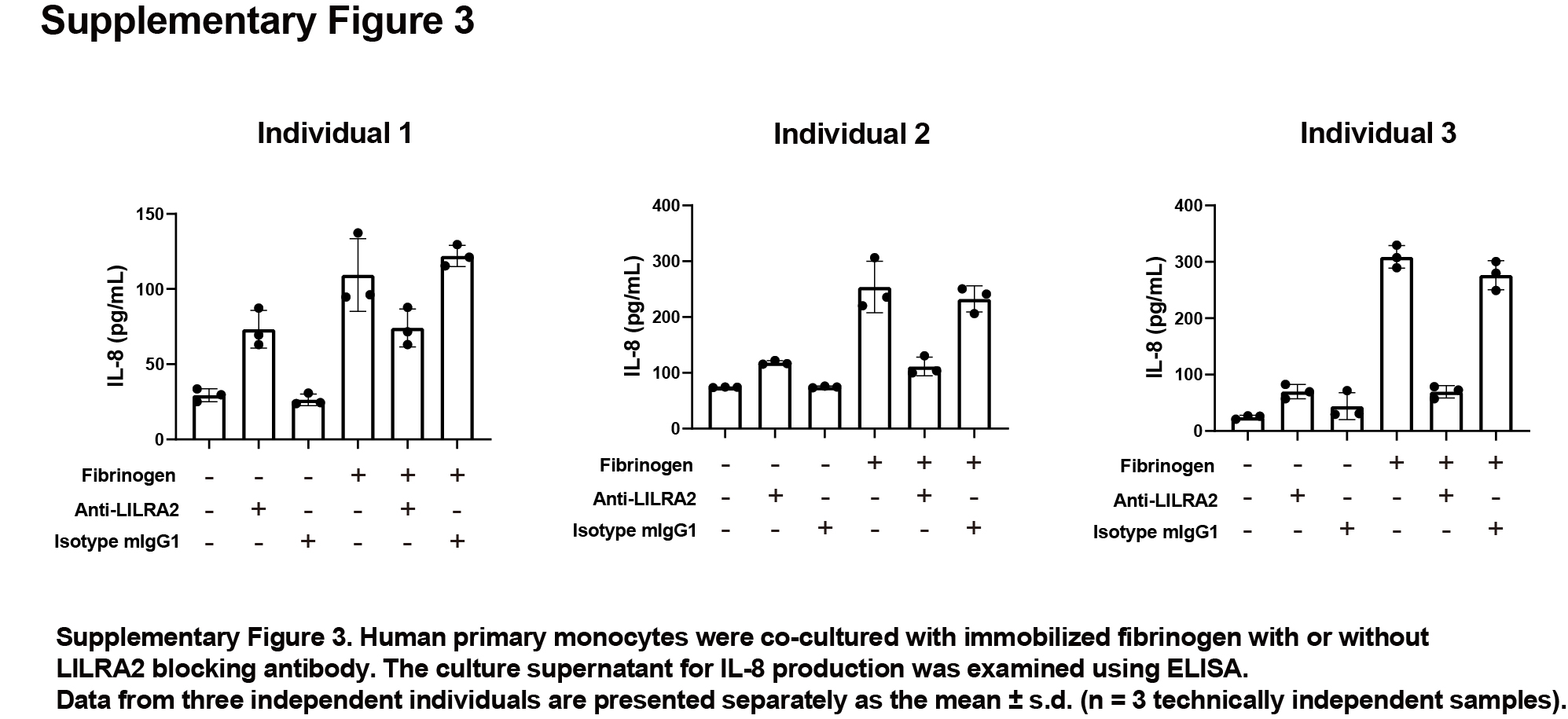

Supplement: Supplementary file 3 [file Image3.jpeg]

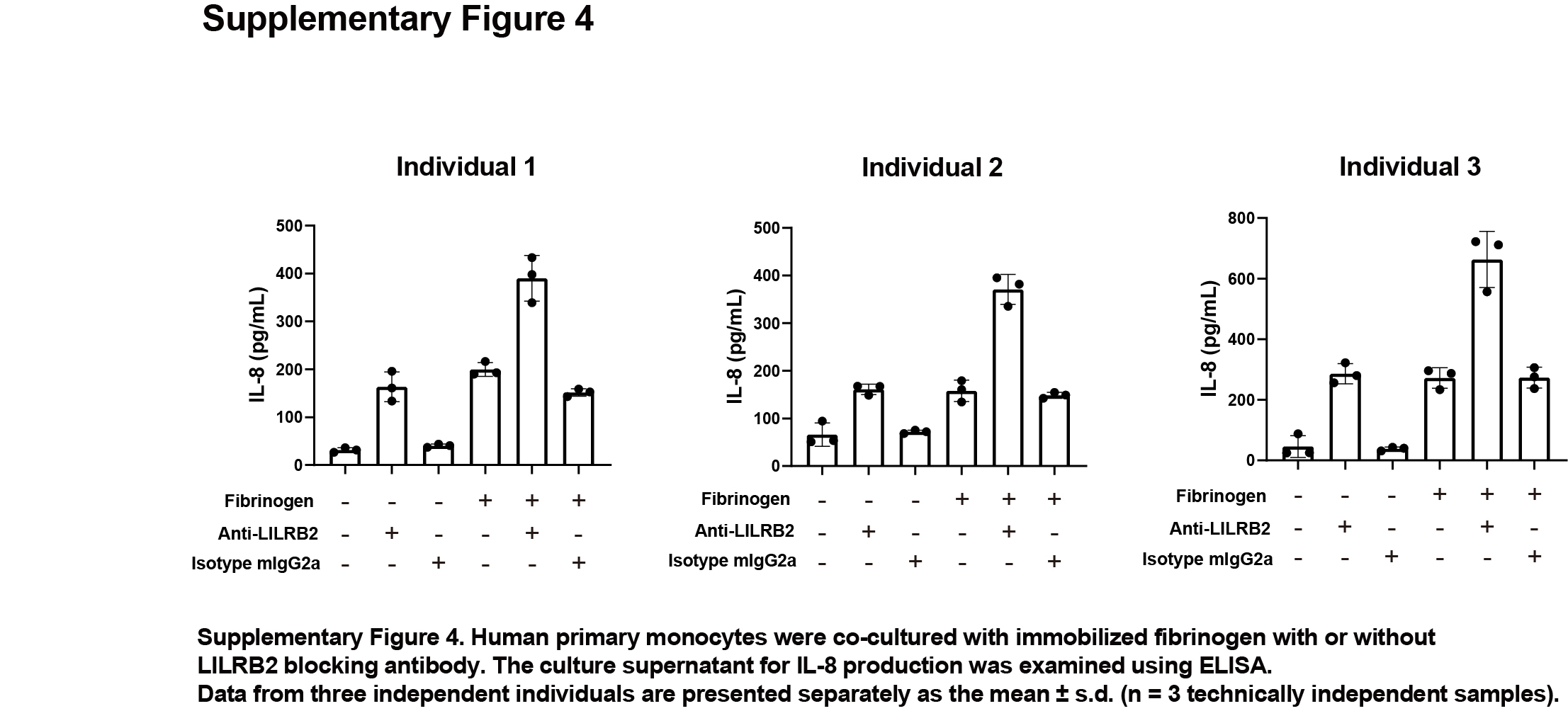

Supplement: Supplementary file 4 [file Image4.jpeg]

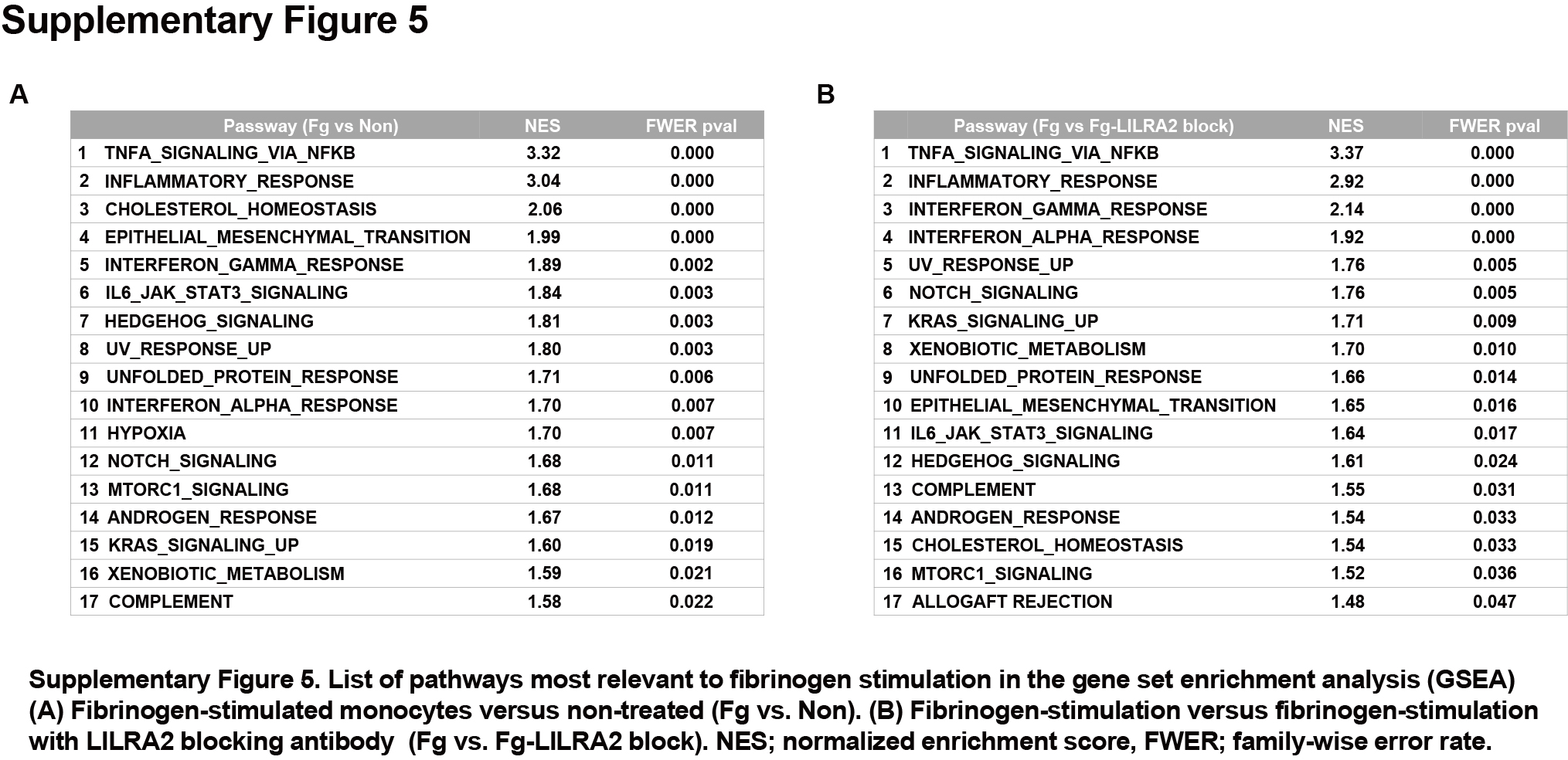

Supplement: Supplementary file 5 [file Image5.jpeg]

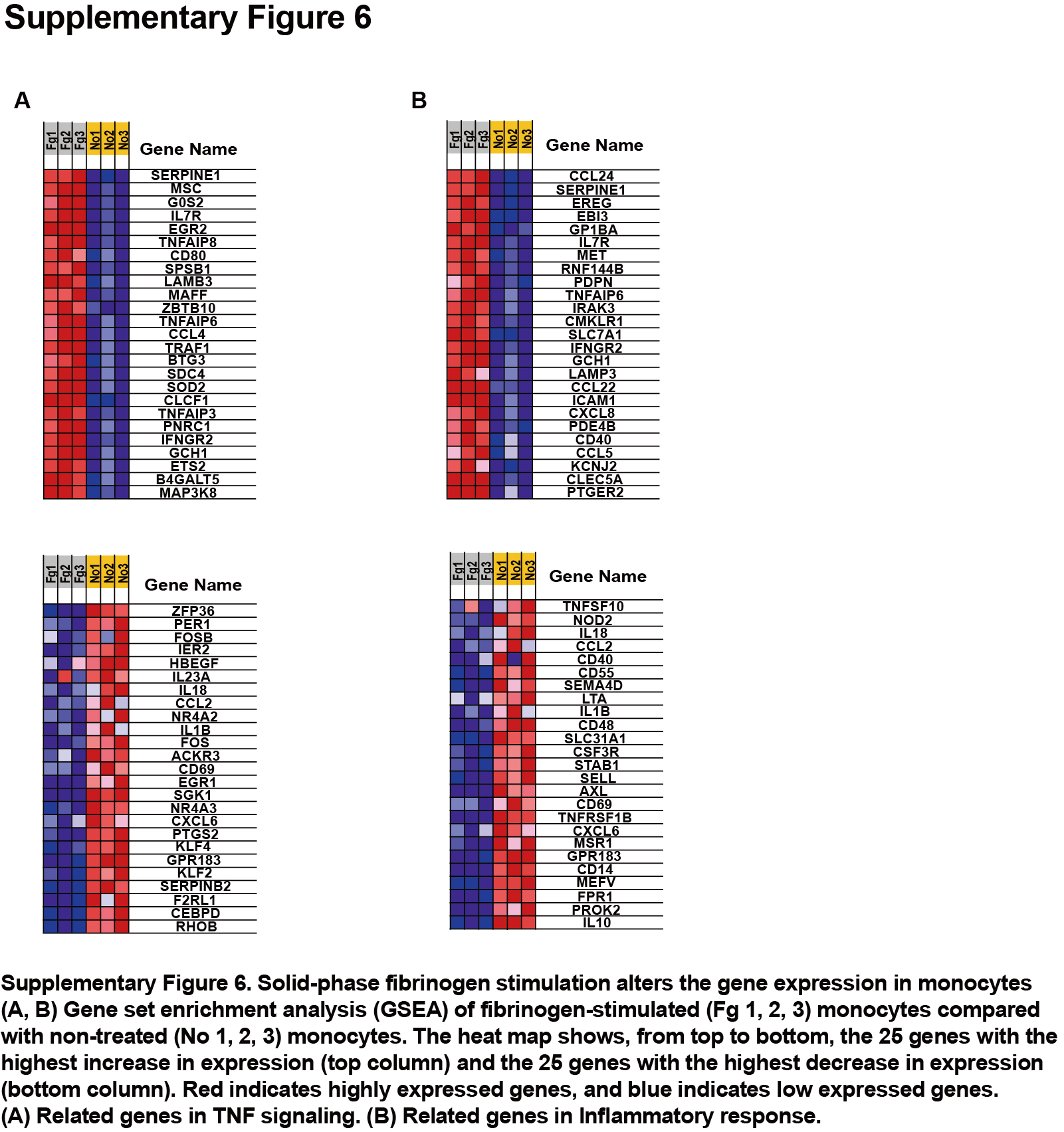

Supplement: Supplementary file 6 [file Image6.jpeg]

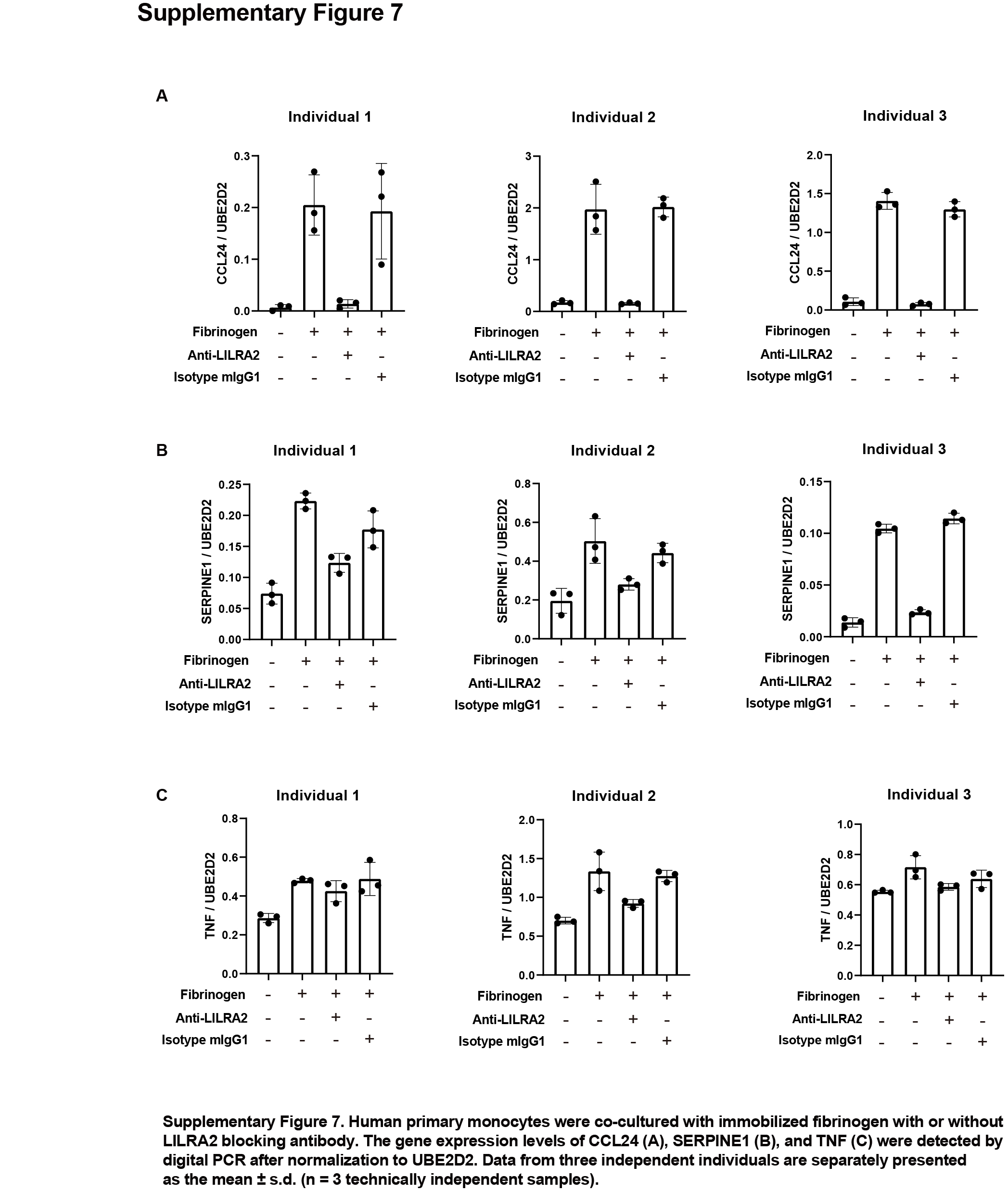

Supplement: Supplementary file 7 [file Image7.jpeg]
